# Supplementary material for: HIVprotI: an integrated web based platform for prediction and design of HIV proteins inhibitors
Source: J Cheminform. 2018 Mar 9;10:12. doi: 10.1186/s13321-018-0266-y (PMC5845081; doi:10.1186/s13321-018-0266-y)
Supplement: Supplementary file 2 — Additional file 2. Source code of HIVProtI web server. [file 13321_2018_266_MOESM2_ESM.zip › HIVprotI_Source-code/hivprotI/weka-3-6-6/doc/weka/gui/DatabaseConnectionDialog.html]

DatabaseConnectionDialog


---


|  |  |  |
| --- | --- | --- |
| **Overview** \| **Package** \| **Class** \| **Tree** \| **Deprecated** \| **Index** \| **Help** \| **Weka's home** | |  |
| **PREV CLASS**   **NEXT CLASS** | **FRAMES**    **NO FRAMES**     **All Classes** |  |
| SUMMARY: NESTED \| FIELD \| CONSTR \| METHOD | DETAIL: FIELD \| CONSTR \| METHOD |  |


---


## weka.gui Class DatabaseConnectionDialog

```
java.lang.Object
  java.awt.Component
      java.awt.Container
          java.awt.Window
              java.awt.Dialog
                  javax.swing.JDialog
                      weka.gui.DatabaseConnectionDialog
```

**All Implemented Interfaces:**: java.awt.image.ImageObserver, java.awt.MenuContainer, java.io.Serializable, javax.accessibility.Accessible, javax.swing.RootPaneContainer, javax.swing.WindowConstants

---

``` public class DatabaseConnectionDialog extends javax.swing.JDialog ```

A dialog to enter URL, username and password for a database connection.

**Version:**
:   $Revision: 7059 $

**Author:**
:   Dale Fletcher (dale@cs.waikato.ac.nz)

**See Also:**: Serialized Form

---

**Nested Class Summary**

**Nested classes/interfaces inherited from class java.awt.Dialog**

`java.awt.Dialog.ModalExclusionType, java.awt.Dialog.ModalityType`

**Nested classes/interfaces inherited from class java.awt.Component**

`java.awt.Component.BaselineResizeBehavior`


**Field Summary**

**Fields inherited from class java.awt.Dialog**

`DEFAULT_MODALITY_TYPE`

**Fields inherited from class java.awt.Component**

`BOTTOM_ALIGNMENT, CENTER_ALIGNMENT, LEFT_ALIGNMENT, RIGHT_ALIGNMENT, TOP_ALIGNMENT`

**Fields inherited from interface javax.swing.WindowConstants**

`DISPOSE_ON_CLOSE, DO_NOTHING_ON_CLOSE, EXIT_ON_CLOSE, HIDE_ON_CLOSE`

**Fields inherited from interface java.awt.image.ImageObserver**

`ABORT, ALLBITS, ERROR, FRAMEBITS, HEIGHT, PROPERTIES, SOMEBITS, WIDTH`


**Constructor Summary**

`DatabaseConnectionDialog(java.awt.Frame parentFrame)`
  
          Create database connection dialog.

`DatabaseConnectionDialog(java.awt.Frame parentFrame,
java.lang.String url,
java.lang.String uname)`
  
          Create database connection dialog.

`DatabaseConnectionDialog(java.awt.Frame parentFrame,
java.lang.String url,
java.lang.String uname,
boolean debug)`
  
          Create database connection dialog.


| **Method Summary** | |
| --- | --- |
| `void` | `DbConnectionDialog(java.lang.String url, java.lang.String uname)`             Display the database connection dialog |
| `void` | `DbConnectionDialog(java.lang.String url, java.lang.String uname, boolean debug)`             Display the database connection dialog |
| `boolean` | `getDebug()`             Returns the debug flag |
| `java.lang.String` | `getPassword()`             Returns password from dialog |
| `int` | `getReturnValue()`             Returns which of OK or cancel was clicked from dialog |
| `java.lang.String` | `getURL()`             Returns URL from dialog |
| `java.lang.String` | `getUsername()`             Returns Username from dialog |
| `static void` | `main(java.lang.String[] args)`             for testing only |

**Methods inherited from class javax.swing.JDialog**

`getAccessibleContext, getContentPane, getDefaultCloseOperation, getGlassPane, getGraphics, getJMenuBar, getLayeredPane, getRootPane, getTransferHandler, isDefaultLookAndFeelDecorated, remove, repaint, setContentPane, setDefaultCloseOperation, setDefaultLookAndFeelDecorated, setGlassPane, setJMenuBar, setLayeredPane, setLayout, setTransferHandler, update`

**Methods inherited from class java.awt.Dialog**

`addNotify, getModalityType, getTitle, hide, isModal, isResizable, isUndecorated, setModal, setModalityType, setResizable, setTitle, setUndecorated, setVisible, show, toBack`

**Methods inherited from class java.awt.Window**

`addPropertyChangeListener, addPropertyChangeListener, addWindowFocusListener, addWindowListener, addWindowStateListener, applyResourceBundle, applyResourceBundle, createBufferStrategy, createBufferStrategy, dispose, getBufferStrategy, getFocusableWindowState, getFocusCycleRootAncestor, getFocusOwner, getFocusTraversalKeys, getGraphicsConfiguration, getIconImages, getInputContext, getListeners, getLocale, getModalExclusionType, getMostRecentFocusOwner, getOwnedWindows, getOwner, getOwnerlessWindows, getToolkit, getWarningString, getWindowFocusListeners, getWindowListeners, getWindows, getWindowStateListeners, isActive, isAlwaysOnTop, isAlwaysOnTopSupported, isFocusableWindow, isFocusCycleRoot, isFocused, isLocationByPlatform, isShowing, pack, paint, postEvent, removeNotify, removeWindowFocusListener, removeWindowListener, removeWindowStateListener, reshape, setAlwaysOnTop, setBounds, setBounds, setCursor, setFocusableWindowState, setFocusCycleRoot, setIconImage, setIconImages, setLocationByPlatform, setLocationRelativeTo, setMinimumSize, setModalExclusionType, setSize, setSize, toFront`

**Methods inherited from class java.awt.Container**

`add, add, add, add, add, addContainerListener, applyComponentOrientation, areFocusTraversalKeysSet, countComponents, deliverEvent, doLayout, findComponentAt, findComponentAt, getAlignmentX, getAlignmentY, getComponent, getComponentAt, getComponentAt, getComponentCount, getComponents, getComponentZOrder, getContainerListeners, getFocusTraversalPolicy, getInsets, getLayout, getMaximumSize, getMinimumSize, getMousePosition, getPreferredSize, insets, invalidate, isAncestorOf, isFocusCycleRoot, isFocusTraversalPolicyProvider, isFocusTraversalPolicySet, layout, list, list, locate, minimumSize, paintComponents, preferredSize, print, printComponents, remove, removeAll, removeContainerListener, setComponentZOrder, setFocusTraversalKeys, setFocusTraversalPolicy, setFocusTraversalPolicyProvider, setFont, transferFocusBackward, transferFocusDownCycle, validate`

**Methods inherited from class java.awt.Component**

`action, add, addComponentListener, addFocusListener, addHierarchyBoundsListener, addHierarchyListener, addInputMethodListener, addKeyListener, addMouseListener, addMouseMotionListener, addMouseWheelListener, bounds, checkImage, checkImage, contains, contains, createImage, createImage, createVolatileImage, createVolatileImage, disable, dispatchEvent, enable, enable, enableInputMethods, firePropertyChange, firePropertyChange, firePropertyChange, firePropertyChange, firePropertyChange, firePropertyChange, getBackground, getBaseline, getBaselineResizeBehavior, getBounds, getBounds, getColorModel, getComponentListeners, getComponentOrientation, getCursor, getDropTarget, getFocusListeners, getFocusTraversalKeysEnabled, getFont, getFontMetrics, getForeground, getHeight, getHierarchyBoundsListeners, getHierarchyListeners, getIgnoreRepaint, getInputMethodListeners, getInputMethodRequests, getKeyListeners, getLocation, getLocation, getLocationOnScreen, getMouseListeners, getMouseMotionListeners, getMousePosition, getMouseWheelListeners, getName, getParent, getPeer, getPropertyChangeListeners, getPropertyChangeListeners, getSize, getSize, getTreeLock, getWidth, getX, getY, gotFocus, handleEvent, hasFocus, imageUpdate, inside, isBackgroundSet, isCursorSet, isDisplayable, isDoubleBuffered, isEnabled, isFocusable, isFocusOwner, isFocusTraversable, isFontSet, isForegroundSet, isLightweight, isMaximumSizeSet, isMinimumSizeSet, isOpaque, isPreferredSizeSet, isValid, isVisible, keyDown, keyUp, list, list, list, location, lostFocus, mouseDown, mouseDrag, mouseEnter, mouseExit, mouseMove, mouseUp, move, nextFocus, paintAll, prepareImage, prepareImage, printAll, remove, removeComponentListener, removeFocusListener, removeHierarchyBoundsListener, removeHierarchyListener, removeInputMethodListener, removeKeyListener, removeMouseListener, removeMouseMotionListener, removeMouseWheelListener, removePropertyChangeListener, removePropertyChangeListener, repaint, repaint, repaint, requestFocus, requestFocusInWindow, resize, resize, setBackground, setComponentOrientation, setDropTarget, setEnabled, setFocusable, setFocusTraversalKeysEnabled, setForeground, setIgnoreRepaint, setLocale, setLocation, setLocation, setMaximumSize, setName, setPreferredSize, show, size, toString, transferFocus, transferFocusUpCycle`

**Methods inherited from class java.lang.Object**

`equals, getClass, hashCode, notify, notifyAll, wait, wait, wait`

**Constructor Detail**

### DatabaseConnectionDialog

```
public DatabaseConnectionDialog(java.awt.Frame parentFrame)
```

:   Create database connection dialog.

    **Parameters:**: `parentFrame` - the parent frame of the dialog

---


### DatabaseConnectionDialog

```
public DatabaseConnectionDialog(java.awt.Frame parentFrame,
                                java.lang.String url,
                                java.lang.String uname)
```

:   Create database connection dialog.

    **Parameters:**: `parentFrame` - the parent frame of the dialog: `url` - initial text for URL field: `uname` - initial text for username field

---


### DatabaseConnectionDialog

```
public DatabaseConnectionDialog(java.awt.Frame parentFrame,
                                java.lang.String url,
                                java.lang.String uname,
                                boolean debug)
```

:   Create database connection dialog.

    **Parameters:**: `parentFrame` - the parent frame of the dialog: `url` - initial text for URL field: `uname` - initial text for username field: `debug` - whether to display the debug checkbox


**Method Detail**

### getURL

```
public java.lang.String getURL()
```

:   Returns URL from dialog

    :   **Returns:**: URL string

---


### getUsername

```
public java.lang.String getUsername()
```

:   Returns Username from dialog

    :   **Returns:**: Username string

---


### getPassword

```
public java.lang.String getPassword()
```

:   Returns password from dialog

    :   **Returns:**: Password string

---


### getDebug

```
public boolean getDebug()
```

:   Returns the debug flag

    :   **Returns:**: true if debugging should be enabled

---


### getReturnValue

```
public int getReturnValue()
```

:   Returns which of OK or cancel was clicked from dialog

    :   **Returns:**: either JOptionPane.OK\_OPTION or JOptionPane.CLOSED\_OPTION

---


### DbConnectionDialog

```
public void DbConnectionDialog(java.lang.String url,
                               java.lang.String uname)
```

:   Display the database connection dialog

    :   **Parameters:**: `url` - initial text for URL field: `uname` - initial text for username field

---


### DbConnectionDialog

```
public void DbConnectionDialog(java.lang.String url,
                               java.lang.String uname,
                               boolean debug)
```

:   Display the database connection dialog

    :   **Parameters:**: `url` - initial text for URL field: `uname` - initial text for username field: `debug` - whether to display the debug checkbox

---


### main

```
public static void main(java.lang.String[] args)
```

:   for testing only


---


|  |  |  |
| --- | --- | --- |
| **Overview** \| **Package** \| **Class** \| **Tree** \| **Deprecated** \| **Index** \| **Help** \| **Weka's home** | |  |
| **PREV CLASS**   **NEXT CLASS** | **FRAMES**    **NO FRAMES**     **All Classes** |  |
| SUMMARY: NESTED \| FIELD \| CONSTR \| METHOD | DETAIL: FIELD \| CONSTR \| METHOD |  |


---
